# 

**Published:** 1885-12

**Authors:** 


### INDEX TO VOLUME LI.

### A.

PAGE

Abdominal Palpation ---------- 288
Abscess of the Liver ---------- 340
Abstracts and Extracts -------- 291
Allaire, P. A., Obituary ------- 399
A Manual of Dermatology, Review ---------------------- 107
American Academy of Siences, ---------------------- 556, 577
American Dermatological Association ---------------------- 374
Anatomical Law -------------- 197
Andrews, Edmund, Clinical Report ---------------------- 68
Arthur, M., Quadruple Pregnancy ---------------------- 151
Atheroma of the Coronary Arteries ---------------------- 41
Atlee, John L., Obituary ------ 486
Auricle, Operation for Deformity of ---------------------- 449
Abdominal Palpitation, Review, 288

### B.

Bacteriotherapy, Extract ------
Bartlett, John, Laceration of the Cervix Uteri ------------ 209, 499
Bettman, Boerne, Blindness ---- 525
Bladder, Paralysis of, Abstract - 493
Bladder, Restriction of, Abstract, 490
Blindness following Hæmorrhage, Bettman ---------------- 525
Bogue, R. G., Nephrectomy --- 45
Books Received ------------ 289, 200
Book Reviews, 93, 193, 284, 393, 478
Bromidrosis of the Feet, Abstract ---------------------- 300
Butterfield, F. A., Correspondence ---------------------- 560

PAGE

Byford, Henry T., Leoi-myoma, ---------------------- 142, 166
Infantile Eczema ---------- 249
Nervous Paroxysm -------- 562

### C.

Caffeine, Poisoning by, Abstract ---------------------- 494
Carcinoma of the Pylorus, Andrews ---------------------- 69
Carter, J. M. G., Tonsilitis -- 514, 571
Cary, Frank, Hospital Report -- 340
Casselberry, W. E., Atresia of Posterior, Nares ------------ 172
Cervix Uteri, Laceration of, Bartlett ------------------- 209
Chemistry, Review ----------- 196
Chemistry, Applied Medical, Review -------------------- 481
Chemistry, Text-Book of, Review ---------------------- 482
Chicago as a Medical Centre, Editorial ------------------ 440
Chicago Gynecological Society, Meeting, May 29, 1885 ------ 58
June 19, 1885 ------ 166
July 17, 1885 ------ 351
Sept. 18, 1885 ------ 356
Oct. 16, 1885 ------- 466
Chicago Medical Society, Meeting, June 15, 1885 ---------- 45
July 6, 1885 ------------ 50
July 20, 1885 ---------- 172
Aug. 3, 1885 ----------- 237
Aug. 17, 1885 ---------- 242
Sept. 21, 1885 ---------- 367
Oct. 5, 1885 ----------- 456
Nov. 4, 1885, ---------- 562
Nov. 16, 1885 ---------- 571

PAGE

Chicago Society of Ophthalmology and Otology, August 11, 449
Children, Diseases of, Review 393
Cholera, Editorial 229
Cholera-Inoculation, Editorial 233
Cholera, Inoculation for 40
Cholera, Treatment of 442
Cholera, Treatise on, Review 195
Cicatrization of Blood-vessels, Review
Clacius, C. E., Syzygium Jambolanum 138
Chemical Reports 68
Cocaine Muriate, Poisoning by, Letter 349
Coleman, W. Franklin, Does Tobacco Impair Sight? 216
Colotomy, Andrews 68
County Provision for the Insane, Kiernan 424
Editorial 439
Cook County Hospital, Report 328
Caronary Arteries, Atheroma of 41
Correspondence, Domestic 162, 234, 347, 560
Correspondence, Foreign 442
Coryza, Acute Treatment of, Robison 52

D.

Davis, C. G., Ovariotomy 312
Diet and Management of Artificially-Fed Infants, Foster 131, 351
Digestive Ferments, Abstract 295
Digitalis 163
Diphtheria, Turpentine in Abstract 494
Diseases of the Eye, Review 102
Does Tobacco Impair Sight? Coleman 216
Dudley, E. C., Abdominal Surgery Abroad 356

E.

Eczema, Infantile, Byford 249
Editorial 37, 158, 228, 342, 439, 532
Eserine, Treatment of Glaucoma, Richey 519
Etheridge, James H., Foetus Papyraceus 113, 166
Exchanges Received 495
Expede Herculem, Editorial 159

PAGE

Experimental Pharmacology, Review 104
Extracts 203, 400, 487

F.

Foetus Compressus 561
Foetus Papyraceus, Etheridge 113, 166
Foster, Addison H, Artificially-Fed Infants 131, 351
Fownes, Chemistry, Review 587

G.

Glaucoma, Treatment of, with Eserine, Richey 519

H.

Hand-Book of Diseases of the Skin, Review 95
Hay-fever; Its Cause and Cure, Ingals 1
Health Department, City of Chicago, Report 253
Hiccough, Remedy for, Extract 400
Hoadley, A. E., Infant-Feeding 144
Hospital Reports 328
Hypnotism, Paoli 505

I.

Illinois State Board of Health
April 16 and 17, 1885 76
July 2 and 3, 1885 185
Infant-feeding, Hoadley 144
Ingals, E. Fletcher, Hay-fever 1
Inoculatiou for Cholera and Yellow Fever 40
Insane, County Provision for, Kiernan 424
International Medical Congress, 153
Editorial 228, 342
Report of Committee 379
Meeting Executive Committee 391
Editorial 441, 532
Intubation of the Larynx, Waxham 401, 456, 511

J.

Johnson, Frank S., Hospital Report 328

### K.

PAGE

Kennicott, G. W., Correspondence ... 350
Kiernan, James G., County Provision for the Insane ... 424

### L.

Lacerations of Cervix Uteri, Bartlett ... 209
Lagorio, A., Foreign Correspondence ... 71, 72, 442
Laparotomy for Gun-shot Wound, Park ... 412, 461
Leio-Myoma of Vagina and Uterus, Byford ... 142, 166
Local News ... 112, 304
Lombard, C. S., Correspondence 236

### M.

Manganese, Oleote of, Martin ... 211
Manual of Nursing, Review ... 398
Martin, Franklin H.,
Oleate of Manganese ... 211
Position of the Uterus ... 23, 58
Palatable Therapeutics ... 565
McSherry, Richard, Obituary ... 486
Medical Dictionary, Review ... 587
Mercy, Hospital Clinic ... 68
Michigan State Board of Health, July 14 ... 188
Michigan State Medical Society 64
Minor Surgical Gynecology, Review ... 287
Modern Therapeutics, Review ... 93
Motions of the Soft Palate, Review ... 284

### N.

Nephrectomy, Bogue ... 45
Nervous Diseases, Introduction to the Study of, Review ... 397
Nervous Paroxysm or Syncope, Byford ...
Newman, Henry P., Shock in Parturition ... 121, 351
News Items ... 111, 197, 303, 399, 482

### O.

Obituary ... 399, 486
Oculo-motor Paralysis, Tilley ... 205
Ohrenheilkunde, Review ... 478
Oleate of Manganese, Martin ... 211

PAGE

On the Treatment of Gonorrhea, Review ... 101
Oral Surgery, Review ... 395
Ovariotomy, Five cases of Davis ... 312

### P.

Palatable Therapeutics, Martin ... 565
Pamphlets Received ... 201, 290
Paoli, G. C., Hypnotism ... 505
Paralysis of the Oculo-Motor, Tilley ... 305
Park, A. V., Laparotomy for Gun-shot wound ... 412, 461
Pathological Anatomy, Review ... 285
Pilocarpin, Ecbolic Action of ... 72
Pharmacology, Review ...
Pharmacology and Therapeutics, Abstract ... 291
Philadelphia Academy of Surgery ... 580
Phthisis Pulmonalis, Review ... 193
Porter, Miles F., Correspondence ... 561
Posterior Nares, Atresia of ... 172
Potter, L. T., Syphilis ... 369
Practical Medicine, Review ... 196
Practical Sanitation ... 203
Primer Lesson in "Metaphysics," Correspondence ...

### Q.

Quadruple Pregnancy, Arthur ... 151

### R.

Remarks on the Toxic Properties of Sassafras, Bartlett ... 499
Reports of the Transactions of the Chicago Medical Societies, Editorial ... 344
Richey, S. O., Treatment of Glaucoma ... 519
Robison, J. A., Acute Coryza ... 52

### S.

Sanitary Convention at Ypsilanti, Michigan ... 176
Sassafras, Toxic Properties of, Bartlett ... 499
Sawyer, E. Warren, Forceps ... 15, 47
Shock and Nervous Influence in Parturition, Newman ... 121, 351

PAGE

Sinclair, Charles F., Special Study ..... 367
Sun-Blindness ..... 451
Smith, Charles Gilman, Correspondence ..... 162
Society Reports, 45, 166, 237, 351, 449, 562.
Special versus General Study in Medicine, Sinclair ..... 367
Spiritualism and Hypnotism, Paoli ..... 505
St. Luke's Hospital, Report ..... 340
Sun-Blindness, Sinclair ..... 451
Syphilis, Treatment of, Potter ..... 369
Syzygium Jambolanum, Clacius ..... 138

T.

Tait, Lawson, on the Modern Treatment of Uterine Myoma, Editorial ..... 231
Text-Book of Practical Medicine, Review ..... 105
Tilley, Robert ..... 50
Oculo-Motor Paralysis ..... 205
The Continued Pelvic Curve in the Obstetric Forceps, Sawyer ..... 15, 47
The Micro-Chemistry of Poisons, Review ..... 99
The Normal Position of the Uterus and its Relation to the Other Pelvic Organs, Martin ..... 23

PAGE

The Oleates, Review ..... 103
Tonsilitis, Report of Two Hundred cases of, Carter ..... 514, 571
Toxicity of Normal Urine, Abstract ..... 298
Traumaticine, Uses of ..... 71
Turpentine in Diphtheria, Abstract ..... 494
Typhoid Fever, Report ..... 328
Tyrotoxicon, Abstract ..... 487

U.

Urinary and Renal Derangements, Review ..... 396

V.

Voice, Song and Speech, Review ..... 110
Verdict of Court-Martial, Abstract ..... 207

W.

Waxham, F. E., Intubation of the Larynx ..... 401, 457, 511
Western Society of Psychical Research ..... 378
"Words and Their Uses," Editorial .....

Y.

Yellow Fever, Inoculation for ..... 40

LIBRARY INDEX S.G.O.

SUBSCRIPTION PRICE, $3.00 PER YEAR.

Whole Number, 312. DECEMBER, 1885. Vol. LI., Number 6.

THE

### CHICAGO MEDICAL JOURNAL AND EXAMINER

EDITORS:

S. J. JONES, M.D., LL.D.

W. W. JAGGARD, A.M., M.D. HAROLD N. MOYER, M.D.

COLLABORATORS.

CHICAGO:

PROFESSOR J. A. ALLEN, M. D., LL. D.
PROFESSOR E. ANDREWS, M. D., LL. D.
PROFESSOR W. H. BYFORD, A. M., M. D.
PROFESSOR O. C. DEWOLF, A. M., M. D.
PROFESSOR E. C. DUDLEY, A. B., M. D.
PROFESSOR J. H. ETHERIDGE, A. M., M. D.
PROFESSOR C. FENGER, M. D.
PROFESSOR J. N. HYDE, A. M., M. D.
PROFESSOR E. F. INGALS, A. M., M.D.
PROFESSOR H. A. JOHNSON, M. D., LL. D.
CHARLES GILMAN SMITH, A. M., M.D.
J. F. TODD, M. D.

MILWAUKEE, WISCONSIN:
PROFESSOR N. SENN, M. D.

WASHINGTON, D. C:
S. O. RICHEY, M. D.

UNITED STATES ARMY:
SURGEON, D. L. HUNTINGTON, M. D.

UNITED STATES NAVY:
MEDICAL DIRECTOR, J. M. BROWNE, M. D.
MEDICAL DIRECTOR, A. L. GIHON, A. M., M. D.

CHICAGO:

